# Efficacy and Safety of Dolutegravir/Lamivudine in Antiretroviral Therapy-Naive People Living With HIV-1 and With High-Level Viremia

**DOI:** 10.1097/QAI.0000000000003600

**Published:** 2025-01-14

**Authors:** Leonardo Calza, Vincenzo Colangeli, Maddalena Giglia, Claudio Rigamonti, Isabella Bon, Silvia Cretella, Pierluigi Viale

**Affiliations:** a Department of Medical and Surgical Sciences, Unit of Infectious Diseases, S.Orsola Hospital, IRCCS Azienda Ospedaliero-Universitaria di Bologna, Bologna, Italy; and; b Department of Medical and Surgical Sciences, Unit of Microbiology, S.Orsola Hospital, IRCCS Azienda Ospedaliero-Universitaria di Bologna, Bologna, Italy.

**Keywords:** dual therapy, viremia, resistance, efficacy, tolerability

## Abstract

**Background:**

Dual regimen dolutegravir/lamivudine (DOL/3TC) showed potent efficacy and favorable safety in both antiretroviral therapy-naive and therapy-experienced patients, but data from real life about naive people with high-level viremia are still lacking.

**Methods:**

We performed a retrospective cohort study of people living with HIV who were naive to antiretroviral therapy, had baseline HIV-1 RNA ranging from 100,000 to 500,000 copies/mL, and initiated DOL/3TC. Virologic efficacy and changes in immunologic parameters after 12 months of treatment were evaluated and compared with highly viremic people living with HIV who started a triple antiretroviral combination.

**Results:**

Inclusion criteria were met by 58 patients with median age of 43.4 years. At baseline, mean HIV RNA was 5.4 log_10_ and mean CD4 T lymphocyte count was 488 cells/mm^3^. HIV RNA <50 copies/mL was obtained in 45 patients (77.6% in the intention-to-treat analysis) after 6 months and in 53 patients (91.4%) after 12 months. Reasons for treatment failure were virologic failure in 2 cases and adverse events in 3 cases. No significant changes in median value of lipids were reported, while there was a not significant increase in body weight (+1.18 kg). Virologic and immunologic response at month 12 in patients on DOL/3TC was comparable with that observed in 50 naive patients with high-level viremia and starting a triple antiretroviral therapy.

**Conclusions:**

In this real-life cohort of naive patients with high-level viremia, DOL/3TC was associated with high virologic efficacy and good tolerability after 12 months, supporting use of this dual regimen also in persons with high initial viremia.

## INTRODUCTION

Combination antiretroviral therapy (cART) recommended in treatment-naive people living with HIV-1 (PLWH) usually comprises 3 active antiretroviral agents from 2 drug classes, and allows them to achieve and maintain long-term virologic suppression in association with a life expectancy comparable with that of the general population. Despite their efficacy, many antiretroviral agents are associated with potential long-term toxicities, including metabolic disorders, cardiovascular disease, osteoporosis, and renal dysfunction, and the increasing prevalence of comorbidities in aging PLWH leads to polypharmacy and a higher risk of drug–drug interactions.^1–5^

Two-drug regimens (2DR) reduce the number of antiretroviral drugs PLWH are exposed and may decrease the risk of long-term toxicities, drug–drug interactions, and costs. The optimal antiretroviral agents in a 2DR should have potent and persistent antiviral activity, high genetic barrier, and good tolerability.

The integrase strand transfer inhibitor (INSTI) dolutegravir is characterized by a potent antiviral activity and a high genetic barrier, and is an optimal candidate for a 2DR in association with the well-tolerated nucleoside reverse transcriptase inhibitor lamivudine. In randomized trials and observational studies, the 2DR dolutegravir/lamivudine (DOL/3TC) has shown noninferior efficacy in comparison with traditional 3-drug regimens (3DR) in both antiretroviral therapy-naive and therapy-experienced PLWH.^6–10^

In GEMINI-1 and GEMINI-2 multicenter, double-blind, randomized trials, the 2DR dolutegravir plus lamivudine was noninferior to 3DR dolutegravir plus tenofovir disoproxil fumarate/emtricitabine after 3 years, showing high barrier to viral resistance and long-term tolerability.^6^ In a systematic review and meta-analysis including 14 randomized controlled trials evaluating the efficacy and/or safety of dolutegravir plus lamivudine in comparison with guideline-recommended 3DR in 10,043 naive PLWH, efficacy and safety outcomes for 48 weeks were similar for 2DR and 3DR.^7^

In randomized trials, proportion of participants with HIV RNA <50 copies/mL obtained with dolutegravir plus lamivudine was comparable with that of 3DR also in patients with baseline HIV RNA ranging from 100,000 to 500,000 copies/mL,^6,7^ but data about virologic efficacy in subjects with high-level viremia at baseline are still lacking in clinical practice.

The aim of our observational study is to evaluate efficacy and safety of 2DR dolutegravir/lamivudine in antiretroviral therapy-naive PLWH with initial high-level HIV viral load.

## METHODS

We performed a single-center, observational, retrospective study of adult PLWH naive to antiretroviral therapy, referred to our Clinic of Infectious Diseases from January, 2020, through December, 2022, with baseline plasma HIV RNA ranging from 100,000 to 500,000 copies/mL, with baseline genotypic testing, and who initiated the daily single-tablet regimen DOL/3TC or a daily single-tablet 3DR. Candidates were those who received at least 1 dose of antiretroviral treatment. All enrolled subjects were followed for 12 months.

Exclusion criteria were having received a previous antiretroviral treatment for >7 days, baseline genotypic testing showing reduced sensitivity to lamivudine or dolutegravir or other drugs included in the antiretroviral combination, chronic hepatitis B virus infection, pregnancy, alcohol abuse or intravenous drug use, alanine aminotransferase (ALT) or aspartate aminotransferase (AST) >80 U/L, creatinine >1.5 mg/dL, and treatment with medications or herbal supplements known to affect the pharmacokinetics of current antiretroviral drugs. Alcohol abuse and intravenous drug dependence were defined as a daily alcohol consumption >30 g and ≥1 intravenous drug use within 6 months before starting the dual regimen, respectively.

Demographic, clinical, and laboratory data were recorded at the start of therapy and at 6-month intervals during the 12-month follow-up. All the available data were collected during routine clinical care. All the plasma samples were analyzed for HIV-RNA level using the automated COBAS AmpliPrep Instrument for specimen processing and the COBAS TaqMan Analyzer for amplification and detection (Roche CobasAmpliPrep/Cobas TaqMan HIV-1 tests version 2.0; Roche Diagnostics, Mannheim, Germany).

In the DOL/3TC group, the primary end point was the virologic success at month 12. Results were evaluated both in intention-to-treat (ITT) and per-protocol (PP) analyses. The ITT analyses included all subjects with at least 1 dose of drug taken and participants who were lost to follow-up, discontinued or changed antiretroviral therapy, died, or had no data at month 12 were considered failures. The PP analysis included all the ITT patients except those with loss of follow-up, who had no 12-month data, or discontinued or changed antiretroviral regimen for reasons other than a virologic failure. The secondary end points included changes in CD4^+^ lymphocyte count, CD4+/CD8+ lymphocyte ratio, metabolic parameters, weight, and body mass index at month 12. Significant weight increase was defined as an increase ≥5% of the baseline weight at month 12.

Virologic success was defined as plasma HIV RNA <50 copies/mL at month 12, and virologic failure as a confirmed plasma HIV RNA ≥50 copies/mL at month 12. Genotypic testing was performed in case of virologic failure. Regarding safety evaluations, the occurrence of adverse events and treatment discontinuation for adverse events were reported by the clinicians in medical records and gathered from their review.

In the 3DR group, only virologic and immunologic responses were evaluated at 12 months and compared with virologic and immunologic efficacy reported in the DOL/3TC group.

The adherence to the current therapy was carefully checked every 6 months on the outpatient visits by self-reported questionnaires. The adopted questionnaire asked how many pills the patients forgot in the past month, in the past week, and in the past 3 days, and led to an evaluation of the treatment adherence as very satisfying (>95%), satisfying (between 85% and 95%), or unsatisfying (<85%). The study was approved by the ethic committee of the S. Orsola-Malpighi Hospital and all participants signed an informed consent after receiving information about the purpose of the study.

### Statistical Analysis

Data are presented as median with interquartile range (IQR) for descriptive data, while comparisons between groups were performed by Student *t* test or Fisher exact test (where appropriate). The significance of changes in all the considered variables was assessed using the paired Student *t* test. Depending on the distribution, the *t* test or Wilcoxon rank-sum test was used to analyze continuous data. All statistical tests were bilateral and a *P* value of <0.05 was considered statistically significant. Excel 2007–2013 (Microsoft, Redmond, WA) was used to input the data, and SPSS 23.0 (IBM, Armonk, NY) was used for statistical analysis.

## RESULTS

Study inclusion criteria were met by 58 patients starting DOL/3TC and 50 starting a 3DR, who were enrolled in the study. In the DOL/3TC group, median age (IQR) was 43.4 (24, 58) years, 49 patients (84.5%) were men, and 55 patients (94.8%) were White. The median baseline log_10_ HIV RNA (IQR) was 5.4 (5.1, 5.6), and 21 patients (36.2%) had HIV RNA ranging from 250,000 to 500,000 copies/mL. Median baseline CD4^+^ lymphocyte count was 488 cells/mm^3^, 21 subjects (36.2%) had a CD4^+^ lymphocyte count <350 cells/mm^3^, and 5 (8.6%) subjects had AIDS diagnosis.

At baseline, 10 patients (17.2%%) had 1 or more comorbidities, and the most common comorbidities were hypertension (10.3% of patients), neuropsychiatric disorders (6.9%), and osteoporosis (6.9%). Notably, we found that the median time from HIV-1 infection confirmation to cART initiation was 6.4 days, and it was less than 10 days in all patients.

Baseline characteristics of patients in the DOL/3TC group were comparable with those of patients in the 3DR group and are summarized in Table 1. In the 3DR group, triple antiretroviral regimen included bictegravir/emtricitabine/tenofovir alafenamide in 26 patients (52%), doravirine/lamivudine/tenofovir disoproxil fumarate in 20 patients (40%), and darunavir/cobicistat/emtricitabine/tenofovir alafenamide in 4 patients (8%).

**TABLE 1.** Baseline Characteristics of the Enrolled Patients

ARV Therapy	DOL/3TC	3DR	*P*
No. of patients	58	50	0.761
Males, no. (%)	49 (84.5)	42 (84)	0.309
White subjects, no. (%)	55 (94.8)	46 (92)	0.288
Age (yr), median (IQR)	43.4 (24, 58)	45.2 (27, 63)	0.178
HIV transmission risk category, no. (%)			
IDU	2 (3)	4 (8)	0.912
MSM	37 (64)	27 (54)	0.461
Heterosexual	19 (33)	19 (38)	0.507
Log_10_ HIV RNA (copies/mL), median (IQR)	5.4 (5.1, 5.6)	5.5 (5.1, 5.7)	0.881
CD4^+^ lymphocyte count (cells/mm^3^), median (IQR)	488 (95, 886)	402 (65, 774)	0.209
CD4^+^/CD8^+^ lymphocyte ratio, median (IQR)	0.54 (0.22, 0.78)	0.56 (0.19, 0.87)	0.176
AIDS diagnosis, no. (%)	5 (8.6)	6 (12)	0.829
Patients with CD4^+^ lymphocyte count <350 cells/mm^3^, no (%)	21 (36.2)	23 (46)	0.105
Patients with CD4^+^ lymphocyte count <200 cells/mm^3^, no (%)	8 (13.8)	6 (12)	0.371
Patients with ≥1 comorbidities, no (%)	10 (17.2)	11 (22)	0.345
Patients with hypertension, no (%)	6 (10.3)	7 (14)	0.682
Weight (kg), median (IQR)	72.4 (61.3, 85.2)	74.1 (58.8, 90.2)	0.438
BMI (kg/m^2^), median (IQR)	23.6 (21.5, 26.3)	23.9 (22.1, 26.8)	0.517
Total cholesterol (mg/dL), median (IQR)	204 (179, 247)	211 (154, 266)	0.198
LDL cholesterol (mg/dL), median (IQR)	139 (102, 168)	142 (94, 181)	0.246
HDL cholesterol (mg/dL), median (IQR)	49 (34, 71)	45 (31, 68)	0.313
Triglycerides (mg/dL), median (IQR)	216 (144, 287)	210 (135, 306)	0.219
Creatinine (mg/dL), median (IQR)	0.91 (0.79, 1.16)	0.82 (0.72, 1.02)	0.527

BMI, body mass index; HDL, high-density lipoprotein cholesterol; IDU, injection drug users; LDL cholesterol, low-density lipoprotein cholesterol; MSM, men who have sex with men.

In the DOL/3TC group, after 6 months of treatment, the proportion of patients with virologic success was 77.6% (45/58) by the ITT analysis and 78.9% (45/57) by the PP analysis. After 12 months, the proportion of patients with virologic success was 91.4% (53/58) by the ITT analysis and 96.4% (53/55) by the PP analysis.

After 12 months, 5 treatment failures (8.6%) were observed: 2 discontinuations (3.4%) due to virologic failure and 3 discontinuations (5.2%) due to nonserious adverse events (Table 2).

**TABLE 2.** Virologic and Immunologic Results at Month 12

ARV Therapy	DOL/3TC	3DR	*P*
No. of enrolled patients	58	50	0.761
Treatment failures, no. (%)			
Discontinuations due to AE	3 (5.2)	2 (4)	0.882
Virologic failures	2 (3.4)	2 (4)	
Missing data	0	0	0.491
Virologic successes (patients with HIV RNA<50 copies/mL)			
ITT analysis, (%)	53/58 (91.4)	46/50 (92)	0.301
PP Analysis (%)	53/55 (96.4)	46/48 (95.8)	0.517
Median change from baseline to month 12 in CD4^+^ lymphocyte count (cells/mm^3^), (IQR)	+145 (+58, +271)	+167 (+62, +302)	0.228
Median change from baseline to month 12 in CD4^+^/CD8^+^ lymphocyte ratio, (IQR)	+0.18 (+0.09, +0.37)	+0.21 (+0.08, +0.42)	0.313

AE, adverse events; IQR, interquartile range; ITT, intention-to-treat; PP, per-protocol.

In the first case of virologic failure, HIV RNA was 660 copies/mL at month 12, and genotypic analysis demonstrated no resistance mutations. This patient restarted antiretroviral therapy with tenofovir alafenamide/emtricitabine/bictegravir and reached a plasma HIV RNA <50 copies/mL within 3 months.

In the second case of virologic failure, HIV RNA was 490 copies/mL at month 12, and genotypic analysis demonstrated no resistance mutations. This patient restarted antiretroviral therapy with tenofovir alafenamide/emtricitabine plus dolutegravir and reached a plasma HIV RNA <50 copies/mL within 3 months.

Patient's adherence to antiretroviral treatment was unsatisfying in both cases of virologic failure. Overall, adherence to medications was very satisfying in 50 out of 58 (86.2%) patients.

There were 3 discontinuations for nonserious adverse events (5.2%): insomnia with sleep disturbances in 1 case (1.7%), headache in 1 case (1.7%), and abdominal pain in 1 case (1.7%).

After 12 months, the median increase (IQR) in CD4^+^ lymphocyte count was +145 cells/mm^3^ (+58, +271) and the number of patients with CD4^+^ lymphocyte count ≥350 cells/mm^3^ increased from 37 (63.8%) to 51 (87.9%). The median increase (IQR) from baseline to month 12 in CD4+/CD8+ lymphocyte ratio was +0.18 (+0.09, +0.37) (Table 2).

In the 3DR group, virologic and immunologic responses at month 12 were comparable with those reported in the DOL/3TC group. The proportion of patients with virologic success was 92% (46/50) by the ITT analysis and 95.8% (46/48) by the PP analysis. Four treatment failures (8%) were observed: 2 discontinuations (4%) due to virologic failure and 2 discontinuations (4%) due to nonserious adverse events. After 12 months, the median increase (IQR) in CD4^+^ lymphocyte count was +167 cells/mm^3^ (+62, +302) and the median increase (IQR) in CD4+/CD8+ lymphocyte ratio was +0.21 (+0.08, +0.42) (*P* = 0.39) (Table 2).

In the DOL/3TC group, during the 12-month follow-up, no significant changes in median concentrations (IQR) of total cholesterol (+16 mg/dL; −7, +28, *P* = 0.711), HDL cholesterol (+0.05 mg/dL; −0.02, +0.08, *P* = 0.817), LDL cholesterol (+8 mg/dL; −4, +15, *P* = 0.605), and triglycerides (+44 mg/dL; −4, +73, *P* = 0.189) were observed.

In terms of kidney and liver function tests, the results demonstrated that changes in median level of both creatinine (+0.15 mg/dL; −0.02, +0.28, *P* = 0.061) and alanine aminotransferase (−3 U/L; −8, + 2, *P* = 0.548) were not significant. At the same time, a slight and not significant increase in median value (IQR) of weight (+1.18 kg; −0.17, + 1.92, *P* = 0.314) and body mass index (+0.28 kg/m^2^; −0.09, + 0.34, *P* = 0.097) was reported at month 12, and no patients had an increase ≥5% in comparison with the baseline weight.

No serious adverse events were reported during the 12-month follow-up, and no grade 3 or 4 clinical events or laboratory abnormalities were recorded in both DOL/3TC and 3DR groups. In the DOL/3TC group, adverse events were described in 11 individuals (18.9%). The most common adverse events were sleeping disturbances (5 patients; 8.6%), diarrhea with abdominal discomfort (3; 5.2%), and headache (3; 5.2%). All reported adverse events were grade 1 or 2.

## DISCUSSION

The antiviral potency of an antiretroviral regimen is carefully predicted by its virologic efficacy in subjects with high-level viremia. In large cohorts of naive PLWH, baseline HIV viral load >100,000 copies/mL is associated with lower rate of virologic suppression and higher risk of viral blips after 1 or 2 years of initial cART.^11,12^ Moreover, the dual regimen ritonavir-boosted darunavir plus raltegravir failed to demonstrate a noninferior efficacy compared with a standard 3DR in individuals with baseline HIV RNA >100,000 copies/mL,^13^ so it is crucial to evaluate the potency of a 2DR in naive patients with high initial viremia.

In our study, initial cART with single-tablet 2DR DOL/3TC was effective in a cohort of 58 PLWH with baseline high-level viremia (HIV RNA ≥100,000 and <500,000 copies/mL), with high rates of virologic suppression (91.4% in the ITT analysis) and a significant increase in median CD4 lymphocyte count (+145 cells/mm^3^) after a 12-month follow-up. Only 2 patients had confirmed virologic failure, but genotype testing showed no resistance-associated mutations for INSTIs or NRTIs.

Moreover, virologic and immunologic efficacy at month 12 was comparable in subjects treated with DOL/3TC and in those with similar baseline viral load and treated with a 3DR.

DOL/3TC showed a favorable safety profile for 12 months of treatment: no serious or grade 3–4 adverse events were reported, and only 3 subjects discontinued treatment because of nonserious adverse events (sleep disturbances, headache, and abdominal pain), while mild neuropsychiatric adverse effects were reported in 8 cases (13.8%).

Starting DOL/3TC did not lead to significant changes in lipid parameters or hepatic and renal function tests, and a small and no significant increase in median body weight was described (+1.18 kg).

To investigate antiviral potency of the 2DR dolutegravir plus lamivudine versus the standard 3DR dolutegravir plus tenofovir disoproxil fumarate/emtricitabine, a post hoc analysis was performed assessing antiviral response rates in the GEMINI-1 and GEMINI-2 randomized trials. In 293 participants with baseline viral load >100,000 copies/mL, median change in viral load from baseline at week 4 was −3.38 and −3.4 log_10_ copies/mL in the 2DR and 3DR groups, respectively. The median reduction in viral load was comparable between groups and was maintained through 48 weeks. At week 24, the proportion of patients with HIV RNA <50 copies/mL was similarly high in both groups, and similar rates of viral suppression were observed in the Snapshot analysis at week 48 in the 2DR and 3DR groups of patients with baseline viremia >100,000 copies/mL (92% and 90%, respectively). No patients with confirmed virologic failure had treatment-emergent INSTI- or NRTI-associated resistance mutations.^14^

A retrospective–prospective observational study enrolled 42 naive PLWH who started DOL/3TC and compared virologic response between 20 subjects with baseline low viral load (<500,000 copies/mL) and 22 with baseline high viral load (≥500,000 copies/mL). At week 48, there was no significant difference in proportions of patients with HIV RNA <50 copies/mL between groups (90% in the low viral load group and 95.5% in the high viral load group), such as changes in CD4^+^ lymphocyte count were similar in both groups.^15^

In the DOLAVI observational, prospective, single-arm study, 88 naive PLWH starting cART with dolutegravir plus lamivudine were enrolled, and 17 had baseline HIV RNA >100,000 copies/mL. At week 48, the follow-up was completed by 14 subjects with high initial viremia and all of them had HIV RNA <50 copies/mL.^16^ A prospective, cohort study compared 140 naive patients who received initial cART of either 2DR dolutegravir plus lamivudine or 3DR efavirenz plus tenofovir disoproxil fumarate plus lamivudine. The study included 26 subjects with baseline viral load >500,000 copies/mL, and at week 48, all patients with high initial viremia had HIV RNA <50 copies/mL in both the 2DR and 3DR groups.^17^ A retrospective multicenter study of PLWH starting DOL/3TC as a first-line regimen included 135 participants, 23 of whom (17%) had baseline HIV RNA >100,000 copies/mL. The proportion of participants with viral suppression at week 48 was 85.2% in the ITT analysis, and all patients with high basal viremia were suppressed and continued treatment with DOL/3TC except 2 who were lost to follow-up.^18^ In a retrospective cohort analysis of 276 naive PLWH who initiated 2DR dolutegravir plus lamivudine or 3DR tenofovir alafenamide/emtricitabine/bictegravir, at week 48 the proportion of patients with virologic suppression was comparable in both groups, and there was no significant difference between groups in virologic efficacy also for 44 subjects with basal viremia >100,000 copies/mL.^19^ In all these studies, no resistance-associated mutations emerged in patients with virologic failure.^15–19^

The 2DR DOL/3TC has been evaluated also as a first-line regimen in a test-and-treat setting for newly diagnosed PLWH. In the STAT multicenter, single-arm, pilot study, 133 subjects initiated DOL/3TC 14 days or less after HIV-1 diagnosis, and 39% of participants had a baseline HIV RNA >100,000 copies/mL. At week 24, 82% of all subjects in the ITT analysis achieved HIV RNA <50 copies/mL, and virologic suppression was obtained in the same percentage of patients with initial viremia >100,000 copies/mL.^20^

It is unknown whether the treatment initiation with a 2DR has a different impact on the immunologic parameters and the CD4/CD8 ratio recovery compared with a 3DR. Using observational data from the Spanish HIV Research Network cohort (CoRIS), 3318 individuals who started 2DR dolutegravir plus lamivudine or a dolutegravir- or bictegravir-based 3DR were evaluated, including 1100 subjects (33.1%) with baseline high-level viremia (≥100,000 copies/mL). At week 48, there were no differences between 2DR and 3DR in the rate of CD4/CD8 ratio normalization, and the CD4/CD8 ratio increase was similar in patients with initial low-level or high-level viremia.^21^

Our work clearly presents several limitations. First, it was a monocentric, observational, retrospective study with a limited sample size. Second, in our study high-level viremia was defined as a HIV RNA ranging from 100,000 to 500,000 copies/mL, because DOL/3TC is contraindicated by the European AIDS Clinical Society Guidelines^2^ in naive patients with HIV RNA >500,000 copies/mL. However, clinical data about efficacy of DOL/3TC as initial regimen with baseline HIV RNA >500,000 copies/mL are lacking, but they should be necessary to define more precisely the efficacy of this 2DR in naive individuals with initial very high viremia. Third, some aspects, such as the control of viral replication in the central nervous system or other reservoirs and the effect on the immune activation markers or peripheral fat, were not evaluated in this study.

To conclude, in clinical practice, the dual combination dolutegravir/lamivudine in a single-tablet regimen represents an effective and safe initial regimen in antiretroviral therapy-naive PLWH with baseline high-level viremia (between 100,000 and 500,000 copies/mL), but larger randomized and observational studies are needed to better evaluate the efficacy of this dual therapy in individuals with initial high and very high HIV viral load.
